# Interrogating the agency of non-profit labour market intermediaries: Casting light on ‘shadow spaces’ through an institutional-relational view

**DOI:** 10.1177/23996544241253706

**Published:** 2024-05-09

**Authors:** Norma M Rantisi, Mostafa Henaway, Deborah Leslie

**Affiliations:** 5618 Concordia University, Montreal, QC, Canada; 5618 Concordia University, Montreal, QC, Canada; 7938 University of Toronto, Toronto, ON, Canada

**Keywords:** Labour market intermediaries, non-profit organizations, shadow state, institutional-relational approach, neoliberal state

## Abstract

In a context of government cutbacks, non-profit labour-market intermediaries are assuming a more significant role in efforts to combat precarious employment. Yet such organizations are still subject to state funding regimes, regulations, oversight and neoliberal logics. As such, some scholars argue that they constitute “shadow state” spaces. In this paper, we move beyond the ‘shadow’ concept, casting light on the ways that different state-non-profit relations shape non-profits’ agency to define and realize their respective mandates. Building on a relational perspective, we hold that links between non-profits and the state are not linear. We complement this perspective with an institutional-relational approach to consider how a non-profit’s distinct institutional configuration (i.e., regulations, funders, and partners) enables or forecloses agency vis-à-vis the state apparatus. Through an examination of two non-profit labour market intermediaries that serve immigrant workers in Montreal/Tio’tia:ke, our analysis lends insight into institutional elements that can enlarge a non-profit organization’s space to maneuver.

## Introduction

The last several decades have witnessed a restructuring in labour markets. The demise of standardized, Fordist mass production - associated with strong unions, government support and full-time, secure employment (at least for white male workers) - has given way to a new ‘flexible’ labour market regime, where employers hire and fire at will. There is a weakening of employment protections, and employers are less likely to invest in training and workforce development (except for a small group of core workers). As a result, there are fewer opportunities for advancement within the firm and less ability to improve wages, working conditions or skills (Zizys, 2011: 12).

This regime is characterized by a bifurcation of the labour market, with highly skilled, well-renumerated work at the top and low paid, precarious jobs at the bottom (Zizys, 2011; Martin, 2011; Benner and Pastor, 2016). Precarious work refers to employment that is insecure, casualized or irregular, poorly paid and lacking in benefits (Kalleberg, 2009; Strauss, 2018; Vosko, 2000). Marginalized groups, such as Indigenous and Black populations, women, youth, and immigrants often find themselves concentrated in precarious jobs (Man, 2004). Apart from the more generalized challenges of possessing the right social networks, work experiences or requisite skills, such groups face racial and gender discrimination that make accessing and navigating an increasingly unstructured labour market difficult (Hira-Friesen, 2018; Man, 2004; Premji et al., 2014).

Alongside the restructuring of labour markets, there have been cut-backs in government support associated with the demise of the welfare state. In an era of neoliberalism, unions are also increasingly under attack. All of these conditions combine to create a “perfect storm,” leaving a gap in labour market regulation (Zizys, 2011).

In this context, a range of labour market intermediaries have moved in to fill the void (Cope, 2001; Martin, 2011; Visser et al., 2017). Labour market intermediaries are actors that match workers with employers, advocate for workers vis-a-vis employers and government, and (re-)regulate labour markets through policy mobilization (Benner, 2003; Visser et al., 2017). Non-profit intermediaries play a particularly important role as labour market intermediaries, as they have extensive ties to the communities in which they are embedded. They work to secure employment and develop skills, but also create safe spaces where disadvantaged workers can support one another (Martin, 2011). Non-profit intermediaries are also involved in struggles that challenge contemporary labour markets (Benner and Pastor, 2016).

With government cutbacks, non-profit intermediaries have taken on functions offloaded by the state. At the same time, many of these organizations are increasingly reliant on state funding to carry out their work, which according to Wolch (1990), places them in the shadow of the state, or when indirectly linked to the state, through links to other non-profits, in the 'shadow of the shadow state' (Gilmore, 2017; Gonzalez Benson, 2022). As such, they are increasingly subject to rapidly changing state mandates. According to this view, the broader state apparatus - and associated neoliberal logics - condition the 'rules of the game' by which many non-profit actors operate (Trudeau, 2008; Wolch, 1990), positioning these actors as part of a “non-profit industrial complex” (NPIC) (Gilmore, 2017).

In this paper, we consider the potential for such organizations to exercise agency by examining two non-profit labour-market intermediaries which emerged in Montreal/Tio’tia:ke in the late 1990s/early 2000s to combat labour precarity and promote socio-economic inclusion. The first is Petites-Mains (PM), a government-funded, work-integration social enterprise that trains immigrant women. The second is the Immigrant Workers Center (IWC), an independent grassroots organization that advocates on behalf of migrant workers. Like most non-profits, these organizations are influenced by the broader state apparatus, but still exercise some agency in defining the scope and nature of their activities. We build on a ‘relational’ approach (Baker and McGuirk, 2021; Trudeau, 2008) that acknowledges this agency, supplementing it with an ‘institutional-relational’ approach (Jessop, 1990), to highlight how an organization’s distinct configuration conditions its ability to function as a relatively autonomous space of action. We argue that each of these labour market intermediaries has distinct organizational and funding structures, with relative advantages and disadvantages for realizing their respective mandates (e.g., providing holistic training and/or advocating for labour market changes) vis-a-vis the state (Gonzalez Benson, 2022; Smith, 2017). An analysis of these cases thus offers insight into elements that can best expand their capacity for maneuver, helping labour market intermediaries to navigate and reconfigure – if not fully escape – the NPIC.

Our analysis draws on 24 interviews conducted with Petites-Mains’ founders, directors, trainers and other employees from 2016 to 2019. Interviews were also conducted with Le Collectif des entreprises d’insertion du Québec (the umbrella organization for work-integration social enterprises in Quebec), government officials and organizations procuring services from PM. Ranging between one and 2 hours in length, interviews were digitally recorded, transcribed and coded according to theme. Site visits were conducted at PM to observe training sessions and facilities. The paper also draws upon information provided in training manuals, the organization’s website and annual reports, news reports and government documents, facilitating a triangulation of data.

Research on the Immigrant Workers Center makes use of many of the same methods as the previous one, including observation, an analysis of the IWC’s website, annual reports, briefs and research publications, as well as press coverage relating to the organization. This case study was conducted by one of the authors, who has been a community organizer with the organization since 2007 and utilizes participant observation, including informal interviews and conversations, as well as personal experience. While direct participant engagement can yield deeper insights into the internal strategies and challenges than the standard interview method, we sought to mitigate the imbalance by relying on multiple research methods to cross-check findings.

In the discussion that follows, we begin with a review of the literature on non-profits and labour market intermediaries, focussing on the potential of these organizations to exercise agency within the broader neoliberal apparatus. We then provide a closer examination of the institutional configuration of the two cases to shift the frame of analysis ‘outside of the shadows’ (Baker and McGuirk, 2021), and to analyze how it shapes their ability to define and attain their objectives. The paper concludes by reflecting on the merits and drawbacks of different configurations and the conceptual and policy lessons suggested by the analysis.

## Non-profit organizations: ‘Shadow state’ formations, or spaces of complex state-civil society relations?

Non-profits have become increasingly significant actors in the landscape of social service provision in North America since the 1980s (Martin, 2011; Smith, 2017). While these organizations have long existed to serve the needs of marginalized populations, governments’ heightened retrenchment- or to use Gilmore’s (2017) term, *abandonment* – of social services provided during the heyday of the Keynesian ‘welfare state’ (1940s to 1970s) has meant that non-profit organizations have greater responsibility for the direct delivery of services, including health, housing, employment and education. Consequently, since the 1990s, studies have sought to theorize the implications of this shift for state– non-profit relations and for the role that non-profits play. Wolch (1990), for instance, uses the term ‘shadow state’ to describe the way community-based organizations increasingly fill the gap created by state cutbacks. The ‘shadow state’ concept refers to more than a heightened responsibility on the part of non-profits for functions formerly delivered by the state. It implies the growing influence of the state over the activities of non-profit organizations, with whom they have official service contracts or funding commitments. The term thus captures the positioning of non-profits ever more centrally within the state’s orbit (Fyfe and Milligan, 2003; Wolch, 1990). According to this perspective, non-profit organizations offer the state ‘ready-to-serve’ actors embedded within civil society, which can contain the worst effects of rollbacks on the part of government, limit crisis and/or quell dissent (Gilmore, 2017; Kivel, 2017). The state’s role, in turn, transitions from that of direct service provider to one of overseer (Gilmore, 2017). Here, the state’s influence is exerted through control over regulations governing non-profits and funding regimes, as well as the terms of service contracts.

According to the ‘shadow state’ view, the role that these organizations play is transformed. As they formally incorporate to access government or foundation funds, they become increasingly technocratic, subject to the state’s terminology and hierarchies, as well as its accounting metrics, service standards and program-specific deliverables (Chouinard and Crooks, 2008; Gilmore, 2017). Forced to operate on small budgets, non-profits are praised for their efficiency and accountability, which Gilmore (2017) equates to the fact that contracts can be terminated at any time. These new arrangements lead to increased professionalization of staff, as well as reduced time and resources for direct engagement with civil society, community building, or the promotion of conscious-raising activities (Pierre, 2009; Trudeau, 2008). This leads to an orientation in which “social rights are significantly circumscribed by the market ethos” (Esping-Andersen, 1998: 127; Pierre, 2009). To the extent that “forms create norms” (Gilmore, 2017: 51), non-profit service providers are said to incorporate the neoliberal logics associated with the downloading (and inadequate funding) of services.

Non-profit organizations constituting the ‘shadow state’ are not the only organizations vulnerable to the neoliberal state’s co-optation (Gonzalez Benson, 2022). An array of other grassroots organizations provide critical services to populations in need, but are not formally tied to the state (through major service contracts or government funding). These organizations rely more heavily on philanthropy, foundations or other non-governmental organizations (Gilmore, 2017). According to Gilmore (2017), grassroots organizations do not directly provide services, but often work with service providers, supporting their clients. These groups have strong commitments to political advocacy and engage in forms of collective organizing, directly challenging state policies that harm or neglect marginalized populations (see also Gonzalez Benson, 2022). Yet, as part of an expanding NPIC, they remain subject to a neoliberal policy regime that is conditioned not only by economic dependencies, but by a broader regulatory context and (ideological) state apparatus that place structural limits on their agency. The apparatus straps an array of these nonprofits to funding relationships, infrastructures, and a tacit set of parameters for viable forms of intervention and opposition that can curtail their ability to maneuver completely outside of either the state or ‘shadow state’ orbit. These groups are said to constitute the *‘shadow of the shadow state’* (Gilmore, 2017: 47; Gonzalez Benson, 2022). Some ultimately formalize their status to widen funding options, but even those that remain unincorporated are often still entangled with ‘third sector’ politics and must position themselves from the regulatory margins, or the oppositional exterior, vis-a-vis the apparatus (Trudeau, 2008: 684; Gonzalez Benson, 2022). According to this view, the shadow is cast wide.

### Non-profit labour market intermediaries as ‘shadows’?

As a subset of the broader non-profit industrial complex, it could be argued that non-profit labour market intermediaries represent proto-typical ‘shadows’. The rise of these intermediaries coincides with the transformation of labour markets. In recent years, traditional welfare models based on a commitment to full employment and universal state supports have been replaced by workfare models, where greater onus is placed on individuals to demonstrate their ‘worthiness of support’ by a willingness to pursue any job - irrespective of the structural challenges faced (Cope, 2001; Peck, 2001; Peck and Theodore, 2000). This shift is directly aligned with the broader neoliberal tendency to download responsibility and risk onto the individual. As noted, within this context of reduced direct state support, lax labour standards and a weakening of organized labour, a range of labour market intermediary actors have come to prominence. This includes an array of private sector intermediaries (such as temporary help agencies, consultant brokerage firms, platform job sites, professional employment agencies), membership-based intermediaries (guilds, unions and professional associations), and public sector intermediaries (educational institutions, workforce development intermediaries), as well as non-profit actors (Benner, 2003; Choudry and Henaway, 2014; Martin, 2011; Pierre, 2009; Theodore et al., 2009; Visser et al., 2017).

Non-profit intermediaries include worker centers, community-based training initiatives and work-integration social enterprises. They play a role in the social regulation of labour markets, from providing skills and resources to sanctioning participant behaviour (Cope, 2001; Theodore et al., 2009; Visser et al., 2017). Relative to other intermediaries, their strong civil society linkages provide a more nuanced understanding of community needs. Yet, as Benner (2003: 625) points out, many of these non-profit intermediaries fall within the orbit of the public sector, since like the ‘shadows’ described above, many rely on government funding and service contracts, while others receive funding or work on projects with government-funded providers. This raises questions about the kinds of state-non-profit entanglements such intermediaries represent and the implications of such entanglements for realizing their mandates. How hegemonic is the state? There is a need to move beyond, or outside of, the concept of ‘shadows’, which risks obscuring rather than illuminating state-non-profit connections by presuming a unilinear or top-down relation.^1^ Recent work (Baker and McGuirk, 2021; DeVerteuil et al., 2020; Power et al., 2021; Trudeau, 2008; Trudeau and Veronis, 2009) challenges this view, calling for a relational approach that advocates closer examination of state-non-profit relations to establish power dynamics and flows of influence. The next section elaborates this perspective, explaining how we contribute to it.

Concepts such as ‘shadow state’ and ‘shadow of the shadow state’ tend to ascribe a hegemonic status to the state in its relations with non-profit organizations. In this vein, the only way for non-profits to reclaim agency is to cut all ties to the state (e.g., see Smith, 2017). A relational perspective, by contrast, views non-profits as situated at the intersection of the state and civil society and thus as more of a liminal, rather than a fixed space, with the potential to shape rather than just simply extend state influence. In this way, non-profits serve as “mediators” rather than mere “conduits” (DeVerteuil et al., 2020: 924; Trudeau, 2008; Trudeau and Veronis, 2009; Baker and McGuirk, 2021). Such a perspective foregrounds how non-profit actors’ ties with the state can assume hybrid forms, with the potential for different power dynamics vis-a-vis different programs and/or in connection to different scales of the state (Trudeau, 2008). As well, due to non-profits’ close connections to civil society, a relational approach highlights how the state relies on these organizations for the realisation and legitimation of programs, which can provide non-profits with a point of leverage (Baker and McGuirk, 2021). The complex, multi-scalar constitution of these organizations therefore positions them as spaces of contradiction, wherein the state’s influence is never complete and the room for maneuver is never fully foreclosed (Power et al., 2021). As liminal spaces, they harbour cracks where the inflection of - and resistance to - state agendas can emerge, suggesting that influence can run in more than one direction (Baker and McGuirk, 2021; DeVerteuil et al., 2020; Murtagh and McFernan, 2015; Power et al., 2021; Trudeau, 2008).

In this paper, we build on this relational perspective. Our point of departure is a recognition of the hybrid, complex nature of state-non-profit relations (Baker and McGuirk, 2021; DeVerteuil et al., 2020; Power et al., 2021). However, we move beyond this view, arguing that the institutional architecture in which the hybrid connections are forged matters. While all such organizations are hybrid, they are not hybrid to the same effect. The specific institutional configurations that constitute a given organization can enable and constrain the kinds of agency that non-profits can exercise (Rantisi and Leslie, 2021).

To build on this view, we draw upon an institutional-relational approach proposed by Jessop (1990) (see also MacLeod, 2001). Jessop applies this approach to understand the contradictory and evolving nature of the state, which serves as a site of struggle. In this view, the state is a complex amalgam rather than unitary, and in this sense, can be considered ‘relational.’ However, there is a recognition of power differentials, as there are factions or classes whose interests are dominant. The plurality of forms is hence a ‘relative’ one, suggesting the need to identify the structure and relations of associated agents (Jessop, 1990; MacLeod, 2001). We extend the institutional-relational approach to non-profit spaces, arguing that non-profits are an amalgam of mandates, programs and relations. However, this complex formation is constituted by uneven relations, particularly vis-a-vis the state and other dominant (economic or political) agents. Relations can be delimited, even if not precisely defined, or subject to ‘contingent necessity’ (Jessop, 1990: 12). Thus, the question of whether and how there is space for maneuvre merits further examination and requires a shift from the abstract to concrete (Jessop, 1990). We hold that each organization is embedded in a distinct constellation of networks in terms of partners and power relations, which condition the scope of possibilities. Certain constellations may facilitate more open and fluid state-civil society assemblages that enlarge the space for agency and allow for direct contestation of the regulatory regime. A key line of inquiry that motivates this study, therefore, is in examining the kinds of agency that the distinct institutional configurations of non-profit labour market intermediaries afford, i.e., under what conditions non-profit spaces can define the ‘rules of the game’.

In what follows, we examine two labour market intermediary organizations that operate within the same regulatory context in Quebec. The case of Quebec is particularly interesting because over the last several decades, relative to other Canadian provinces, the government has sought to temper a top-down approach by cultivating a ‘partnership’ model that is more open to civil society and third sector participation in policy construction and implementation (Vaillancourt, 2012). The next section provides a brief overview of this model.

## The rise of the Quebec’s state-non-profit entanglement

Deindustrialization and rising unemployment sparked a process of community mobilization and a series of anti-poverty, anti-racism and feminist social movements in Quebec in the 1980s and 1990s. These campaigns called for a more inclusive democratic structure and for community economic development initiatives focussed on training and job creation, particularly in marginalized communities (Fontan et al., 2006, 2009). As a consequence of these struggles, there was a reconfiguration of the Quebec state to embrace more *plural* logics, including neoliberal regulation, but also neo-welfarist and solidarity-based governance (Vaillancourt, 2012). Within this new structure, community-based organizations emerged as key actors, demanding new institutional and financial arrangements, as well as a distinct identity, autonomy and partnership role with the state (Vaillancourt, 2012, 2015).

Among the changes made, the province created the Secrétariat à l’action communautaire autonome et aux initiatives sociales or SACAIS (a provincial government policy on community action)*,* with the mandate of **“**acknowledging, promoting, and consolidating independent community action, more specifically rights advocacy organizations” (https://www.mtess.gouv.qc.ca/sacais/). This secretariat provides funding to community organizations, including long-term funding. It includes these organizations as “co-builders of policy,” rather than simply “co-providers” (Vaillancourt, 2015: 88). In contrast to the more neoliberal edicts, this particular pool of funding is not directly tied to either state directives or the market, providing some autonomy. However, a key challenge is that the number of recipient organizations is limited (Interviews).

Another change initiated in this time involved greater support for the social economy. The government held two summits in the second half of the 1990s, inviting business leaders, unions, civil society and third sector organizations (Vaillancourt, 2015). A working group was created, and the province set aside funding for the social economy, and in particular, for ‘entreprises d’insertion’ - or what are referred to as ‘work integration social enterprises’ (WISEs) (Interviews; Dolbel, 2009: 3; Cooney, 2011). Government funding included services related to networking, consulting and finance (Mendell, 2009).

While these changes prompted a greater opening for community-based and social economy organizations in policy formation, over time, some organizations developed strong ties to the state through formal contracts (Fontan et al., 2006). As Vaillancourt (2015: 88) argues, despite their agency, many community groups remained in a fragile and asymmetrical relationship with the state.

Our two case studies represent organizations that emerged within this context. Both organizations can be classified as ‘shadow spaces’. However, they are not subject to state influence to the same degree and are differently situated in relation to the state. One organization has direct and formal ties as a service provider – or what Wolch (1990) would call the ‘shadow state’ – whereby the state represents a key institutional anchor. The second operates at a more grassroots-level and is influenced by the state in indirect ways – or what Gilmore (2017) and Gonzalez Benson (2022) call the ‘shadow of the shadow state’. Casting light on both ‘shadows’ is instructive, as most studies have centered on non-profit groups with direct ties (cf. Gonzalez Benson, 2022; Trudeau, 2008). Thus, a more nuanced examination of state-non-profit entanglement for grassroots organizations is warranted (Gonzalez Benson, 2022). In what follows, we take a closer look at the distinct structures and mandates of each organization, and the implications that these have for agency.

## Direct state-non-profit entanglement: The case of Petites mains

### History

Petites-Mains (PM) was established through community mobilization, after a food bank in a predominantly immigrant neighbourhood closed. Most of the recipients at the food bank were immigrant women. In collaboration with a local church, they decided to create a program that focussed on training in industrial sewing (later expanded to include restaurant and office work) (Interviews).

The organization began offering services in 1994, relying mostly on charitable donations and volunteer labour, and generating a small amount of revenue from the sale of goods. In 1995, it registered as a non-profit charity. Initially, there was little government support or interest in the organization’s programming, as garment manufacturing was viewed as a sunset industry (Interviews). However, the organization developed partnerships with local manufacturers, and owing to its successful placements, government support increased over time. This led to an enhanced training curriculum by 1998.

In 2000, the organization registered as an ‘entreprise d’insertion’, and became an official member of an umbrella group, Le Collectif des entreprises d’insertion du Québec (https://www.petitesmains.com/about-us). In Quebec, this designation means that the organization has social economy status and a dedicated stream of funding for its training program through a service contract with Emploi Québec (‘Employment Quebec’), a unit within the Ministry of Labour, Employment and Social Solidarity. The designation brings PM squarely within the state’s orbit (Interviews).

From its earliest days, the organization offered training, but also *accompaniment* - long-term social support tackling the diverse, often structural, challenges that an individual confronts. This involves linking participants to service providers (including family, healthcare and housing services), offering counselling and language instruction. PM also hosts workshops on tenant, labour and civil rights. Its core mandate is to provide a holistic (economic and socio-political) approach to training (Interviews).

The organization has three key objectives. The first is to provide professional training in industrial sewing, office reception and kitchen work. To this end, PM offers hands-on work experience, developed in consultation with its partners and employers. The second goal is to empower women to escape social isolation and exclusion. In relation to this goal, PM emphasizes individualized supports, but also seeks to influence policy agendas relating to precarity. The third aim is to assist women to end dependence on government support, including welfare (Interviews, https://www.petitesmains.com/about-us). As a member of the Collectif, Petites-Mains must adhere to specific criteria to retain its status, including holistic training and partnering with community organizations (https://collectif.qc.ca/criteres).

The focus of the program is on immigrant women who are on social assistance or without income (Interviews). In contrast to traditional workfare programs**,** the training lasts 6 months and participants are paid full-time at minimum wage. Training consists of a variety of hard and soft skills (such as time management, teamwork, budgeting, communication, and independence). The organization also offers job search support and placement services, as well as French language training, and provides follow-up for at least 2 years (Interview, PM representative). PM strives to create “a space of care, ethics and belonging” (DeVerteuil et al., 2020: 922), hosting social events to build ties among participants as a basis for longer-term and collective (rather than merely individualized) forms of support. While the 6-month training serves as the core of their operations, the organization also offers pre-employment services and language classes to a wider clientele of visible minority and immigrant women (Petites-Mains, 2019-2020).

### Funding and partnerships

Today, PM relies heavily on government funding, with Emploi Québec (EQ) covering the participants’ salary for 6 months. Petites-Mains also receives program-specific funds from the federal and city governments for pre-employability and language programs (Petites-Mains, 2019, 2020). Government funding accounts for nearly 45-50% of the organization’s revenues (Interviews). In a context of fluctuating government mandates, PM seeks to generate its own revenue through the sale of goods and services (Interviews). This source of funding accounts for roughly 40% of total revenue and provides some autonomy to compensate for cuts in certain programs or restrictions associated with certain pools of public funds. The bulk of buyers tend to be other non-profit organizations, public institutions (e.g., universities and municipal government) and independent businesses that value PM’s mission (Petites-Mains, 2020; Petites-Mains, 2021; Interviews with buyers). The organization also takes donations from individuals and corporate donors, as well as foundations (https://www.petitesmains.com/fundraiser). It also accesses financing from accredited social economy institutions (Interview, PM representative).

In terms of placements, in the early days, most partnerships were with large manufacturers in the city. However, over time, many manufacturers downsized or shifted offshore (Interviews). Some participants experienced exploitative conditions in these companies (Interviews). As a consequence, greater emphasis has been placed on partnering with local, independent producers, other social enterprises, and public sector actors. These partnerships continue to be shaped by participant feedback (Interview, Petites Mains representative).

The Collectif represents a significant partner. As a federated organization, it represents 49 insertion enterprises across Quebec (https://collectif.qc.ca/), providing resources, training and information on best practices. It creates the space, through annual meetings and workshops, for member organizations to network. The Collectif partners with public sector actors, social economy institutions and community organizations concerned with issues of employment, poverty and exclusion to engage in lobbying and policy debates (Petites-Mains, 2020-2021; Interview, Collectif representative).

Finally, while PM is located in the Villeray-Parc Extension neighbourhood, its relations are not spatially bound. It is part of neighbourhood and city-wide roundtable groups (focussed on the Park-Extension neighbourhood, women, immigrants, refugees, food security, language training and employment), which aim to enhance democratic governance and combat exclusion (Petites-Mains, 2020, 2021). These groups provide a forum, bringing together local community, non-profit and public sector actors (Interview, PM Representative; see also DeVerteuil et al., 2020: 928). These ‘tables’ have regular meetings to identify pressing issues and exchange information and resources.

### Strategies for addressing a socio-political mandate

Owing to its service role, most of PM’s activities are oriented to upgrading skills and individual counselling, rather than broader forms of community-building or advocacy. However, PM hosts workshops on tenant, labour and civil rights to raise awareness of systemic racism and exclusion. This consciousness-raising activity is a critical aspect of empowerment, positioning these issues as collective challenges (Iverson and James, 2014). These workshops link participants to groups engaging in direct forms of advocacy (since the workshops are generally given by organizations such as IWC). And as noted above, PM representatives sit on local roundtables that promote more inclusive governance. Their participation presents opportunities to share knowledge about the challenges participants face. These roundtables also help identify where support is needed, in collaboration with independent community organizations.

PM has also participated in two government committees: a federal advisory committee to develop Canada’s first Poverty Reduction Strategy, and the city’s advisory committee on social solidarity (Annual Reports, 2018-2019, 2020-2021). The Poverty Reduction Strategy was launched in 2019 and includes an enhanced Worker’s Benefit and an income supplement for those precariously employed (Government of Canada, 2018).

Apart from these government-supported channels, advocacy occurs indirectly through the Collectif. The Collectif works in partnership with provincial government bodies, yet has some distance from government due to its ability to finance most activities through member fees (Interviews, PM and Collectif representatives). This status enables it to publish critical position statements on poverty and workfare, to which enterprises such as PM contribute. In 2019, for instance, the Collectif published a document calling on the provincial government to recognize insertion enterprises as ‘community action’ actors rather than ‘service providers’ and provide adequate funding (https://collectif.qc.ca/les-publications/memoires/). The Collectif is also part of an independent anti-poverty coalition called Le Collectif pour un Québec sans pauvreté (or ‘Collective for a Quebec without Poverty’), which in 2020, issued a critique of the provincial government’s punitive measures towards work integration (Interviews, PM and Collectif, https://collectif.qc.ca/les-publications/recherches-et-etudes/). While this action did not lead to an alteration of policy, initiatives such as this help build public awareness and foster network connections that serve as a foundation on which future struggles are waged (Interviews). Through such channels, PM can indirectly - as well as directly - influence policy development.

### Challenges

The organization’s funding and strong public sector partnerships offer it relative stability when compared to many non-profits. But this institutional configuration, where the ‘state’ is a key anchor, also presents constraints. The funding from EQ is aimed at skills training and individual accompaniment. Funding is provided for a number of ‘places’ (i.e., enrolled participants), based on a unit cost per participant (Interview, EQ representative). Acquiring approval for an increase in the number of places can take time and there are long waiting lists (Interview, PM representative; Facebook comments).

Another problem is that the funding from EQ is ‘program-specific’ rather than ‘core,’ which reduces PM’s flexibility in program offering and resource allocation. PM must renew their contract every 3 years and renewal is not guaranteed. As one representative notes, “if you’ve had a poor placement rate or if there have been changes in personnel, they can choose to renew your contract for only a year. And every 10 years, you have to go through a recertification process” (Interview). Owing to its direct and significant ties to the state, the enterprise must continually legitimate its programs, framing them within government priorities and metrics centered on facilitating employment insertion (measured by post-training placement rates) (Interview, EQ official). These metrics do not capture the range of socio-political supports provided, i.e. the “hidden” labour (Martin, 2014). The administrative work in meeting such demands - along with the growth of the business component to address funding shortfalls - crowds out time and resources for other activities, such as community engagement or hosting rights-based workshops (Interviews).

Apart from these challenges, government funding makes PM vulnerable to shifting mandates. While historically PM targeted participants who had little to no paid work experience or income (i.e. those furthest from the labour market and not eligible for social assistance or employment insurance), new government priorities are altering this focus. As the organization explains,

Emploi Québec asked us to prioritize people on social aid or employment insurance. So 60% of our clientele have to be on social assistance or employment insurance…So it’s harder. We have to adapt our interventions because people who have been on social assistance for 10-12 years have different needs… (Interview).

PM also faced unexpected funding cuts by the provincial Ministry of Immigration for accompaniment services for new immigrants (Petites-Mains, 2018). Austerity measures geared to reducing welfare obligations constrain the ability to serve those most in need.

Similar dynamics play out at other scales. After the federal Conservative Party assumed office in 2006, they decided to focus on participants closer to the labour market. This entailed changes to a pre-employment internship program:

they wanted [participants] to quickly enter the job market — to be productive, to pay taxes, to reduce welfare rates….Instead of doing internships, we (place them in) ‘job experiences’ (Interview).

Over time, there has been a noticeable expansion of pre-employment and language classes catering to those closer to the job market (PM Annual Reports, 2017-2018, 2018-2019, 2019-2020). Such trends point not only to the challenges to meeting a hybrid mandate of economic and social inclusion that stem from narrowly defined programming, but also to the challenges associated with funding form (program vs core) and participant eligibility (both in terms of number and kind) (Trudeau, 2008).

## Indirect state-non-profit entanglement: The case of IWC

### History

The Immigrant Workers Centre (IWC) was founded in 2000 with the aim of promoting the rights of precarious workers outside the scope of traditional trade union organizing (Choudry and Henaway, 2012). IWC was built upon - and combines - the traditions of both community and labour organizing. Its unique approach was to construct a labour organization embedded within the community and to create a safe space for immigrant workers to defend their rights (Choudry and Henaway, 2012). Its physical location within the low-income, migrant neighbourhood of Cote-des-Neiges is reflective of this aim.

In the beginning, IWC worked mostly with workers from the Philippines, employed in the clothing industry and care work. It confronted processes of neoliberalization, including trade liberalization and the shift of garment manufacturing offshore (Henaway and Rantisi, 2021). This led to a loss of employment for garment workers, many of whom were immigrants who had worked in the industry for up to 30 years. In 2007, for example, workers from Lamour Inc., a Montreal sock and apparel manufacturer, approached IWC seeking a severance package from the company. Using a worker-led approach, IWC initiated a campaign seeking legal support for workers. Through collective decision-making and direct action, by 2008, the campaign grew to over 100 workers, prompting IWC to shift its priorities from a focus on workplace conditions to severance and retraining packages for laid-off workers. The campaign served as an example of IWC’s flexibility, highlighting its ability to realign its focus to challenges faced by immigrant workers.

Also, during this period, IWC confronted the rise of non-standard employment, which went hand-in- hand with the growth of for-profit temporary placement agencies (Choudry and Henaway, 2014; Vosko, 2000). There was also the rise of Canada’s Temporary Foreign Worker Program. Consequently, IWC created two workers’ committees in 2013: the Temporary Agency Workers’ Association (TAWA) and the Temporary Foreign Workers’ Association (TFWA).

The operations of the Centre currently revolve around four axes. The first includes services and advocacy on behalf of individual workers, including accompanying workers to the Labour Standards Commission to file complaints, providing referrals and liaising with unions**.** The second function is political education for workers, community groups and allies, such as labour rights workshops, political discussions and the ‘skills for change program’ (language and computer literacy training infused with labour and immigration rights). In recognition that individual accompaniment or casework is not enough to alter the structural nature of precarity, the third axis involves building collective campaigns (relating to workplace issues, minimum wage, temporary work or immigration policy). The fourth aim is building community by providing physical space for workers’ committees to coordinate worker and neighbourhood-based campaigns (e.g., International Women’s Day and International Workers’ Day events).

### Funding and partnerships

The IWC’s funding structure has been precarious since the beginning. IWC is a non-profit, rather than a ‘non-profit charity’. This is because charity status restricts political activities (e.g., criticizing a political party). Today, the IWC relies primarily on three sources of funding: individual donations (including academic research grants), union support (through solidarity or project-based funds), foundations and to a limited extent, state bodies. One key foundation is Fondation Beati (associated with the Catholic Church), which has a mandate to support social justice organizations that cannot access traditional streams of funding. This funding has allowed the IWC to work on a variety of projects, such as documenting warehouse working conditions and organizing non-status migrant women. IWC also receives funding from LUSH cosmetics (through its charity arm), which has enabled it to mount an education and outreach project with temporary foreign workers outside the Montreal/Tio’tia:ke region. As IWC has grown, acquiring more institutionalized knowledge and expanding its network, its donations have grown significantly. Limited state funding comes from sources such as the Bureau d’intégration des nouveaux arrivants (‘Office of Integration for New Arrivals’) at the municipal level, which is project-based.

Through consistent mobilization, the Centre has built strong alliances with the established community sector, particularly progressive movements. These partnerships help build a broad base to support members, and they also amplify IWC’s socio-political demands. One key partnership is with the Front de défense de non-syndiqué-es (FDNS). FDNS is a coalition that includes Au bas de l’échelle (a francophone migrant workers’ centre), as well as churches, trade unions, and social movements. This partnership works to improve the conditions of non-unionized workers. Outside of this coalition, IWC works directly with the trade union movement to support IWC projects and address the growing crisis confronted by unions amidst the rise of non-standard employment (Fine, 2006). In contrast to PM, it is the coalition and union movement that serves as the organization’s key institutional anchor.

One union that the IWC works closely with is the left-leaning Conseil Syndicats Nationaux (CSN) Metropolitain Montréal Central Council. CSN supported the garment worker campaign in 2007. In 2013, the IWC and CSN formalized their partnership, supporting the work of TAWA, including the printing of its newspaper *Migrant Voices*. Today, CSN continues to provide support and now has a seat on the IWC board. Other prominent trade union partners include UNIFOR and the Canadian Union of Public Employees.

In addition to unions, IWC has established ties with a variety of community-based organizations – at both the local and national scales – such as those mobilizing around migrant rights, tenant advocacy and housing justice. For example, the IWC is a member of the Migrant Rights Network, comprised of forty-two migrant-led organizations across Canada. This network advocates for full regularization and for annual pan-Canadian ‘days of action’ (https://migrantrights.ca).

Beyond civil society, the IWC also establishes direct and indirect connections with state institutions. For instance, it participates in the Coalition against Precarious Work that brings together domestic workers, Mexicans United for Regularization, TAWA and TFWA. Through its involvement in this coalition, the IWC met with the Ministry of Labour in 2017 to raise workers’ demands concerning precarious work. In 2016, its worker-led branch, TAWA, partnered with the Department of Public Health in Montreal, to author a government-supported report entitled, *Invisible Workers: The Health and Safety of Temporary Agency Workers* which prompted the director of Public Health to make a case for reforms in temporary placement agencies. This partnership also paved the way for IWC to access tripartite bodies (provincial government agencies, employer organizations and civil society actors) relating to health and safety. The IWC’s work with government institutions remains tenuous, but tangible gains have been realized.

In contrast to PM, state bodies are one among other more central partners, and thus, the sphere of direct influence of the state is limited. A primary basis for state-non-profit entanglement, however, is an indirect one. IWC seeks to contest exploitative practices and alter the broader regulatory context that conditions precarity for temporary migrant workers. Thus, a significant part of their work involves an adversarial positioning vis-a-vis the state, a theme to which we turn in the next section.

### Realizing a socio-political mandate through an adversarial form of state-non-profit entanglement

As noted above, IWC engages in advocacy to confront the exploitative conditions that migrant workers face through both individual casework (e.g., filing a complaint on behalf of a worker) and collective forms of organizing (e.g., a campaign that targets an employer). Apart from addressing the individual and collective conditions that migrant workers face, IWC links these conditions to broader systemic issues. For instance, in an attempt to address the structural issues faced by temporary workers, in 2017, TAWA initiated a Stability and Dignity campaign. Four demands were presented to the Minister of Labour, including basic rights to permanent work for all workers, as well as equal access to health and safety. Access to labour rights and state services regardless of immigration status and a minimum wage of $15 an hour were also demanded (TAWA, 2021).

This socio-political advocacy involves direct action (often informed by research conducted with academics, as well as by some public-sector agencies). This action can take the form of flyering, press conferences or protests. In contrast to a service-led approach that seeks to combat precarity primarily within state structures and existing legal frameworks, the IWC’s relative independence allows it to engage more directly in advocacy, applying pressure from outside of the state and ‘shadow state.’ This has led to a number of victories, ranging from successful campaigns against exploitative employers to changes in provincial policy. One example is a campaign that was initiated in 2011 in collaboration with Au bas de l’échelle to raise awareness of the role of temporary agencies in undermining worker rights. The campaign involved direct action by workers and allies, including protests, a bus tour, letter writing and lobbying key government ministries to make temp agencies more accountable for working conditions. Two hundred community organizations and two political parties signed on to demands for tighter regulations on temporary placement agencies.

Another example is the mobilization that led to the passing of Bill 176 in 2019, which requires that temporary placement agencies register with the Ministry of Labour, share responsibility with client companies for employment conditions and provide equal pay for equal work regardless of employment status. This bill was the first major reform of labour standards in Quebec since 2003, illustrating the critical role that IWC plays in moulding the labour market.

In all of its advocacy work, IWC facilitates the active engagement of immigrant workers. IWC creates a collective means for change through the creation of a safe space, where workers can discuss their situations and find creative solutions. Through this engagement, workers are trained to meet with politicians, gain skills in public speaking, hold a press conference and mount campaigns. The goal is consciousness-raising and collective mobilization that is oriented toward confronting the broader structures that produce precarity. This is significant because this population is highly vulnerable and not always able to challenge conditions individually (Hanley and Shragge, 2009). Such an approach departs from a traditional model of union organizing, often characterized by a top-down logic and lacking attention to issues of ethnicity and race. This approach also deviates from a model of community service, where there is a ‘professionalization’ of service provision and a disempowering of participants. While a service-led approach combats precarity within existing state structures and legal frameworks, IWC’s widened space of engagement enables it to apply pressure from the outside. This demonstrates the non-linear nature of non-profit-state relations (Trudeau, 2008).

### Challenges

Independent, non-charity status enables a non-profit like IWC to maintain a circumscribed relation to the state. However, it also limits the pool of resources that the Center can access. This constraint is compounded by the fact that the majority of funding for non-profit organizations is geared towards service provision, which is marginal to IWC’s overall mandate. While there is ample state funding for language courses and integration services, there is little support for community building or collective political mobilization. Another limitation is that state funding emphasizes services for immigrants with status and the IWC’s mission is to support immigrants *irrespective* of status, including temporary and undocumented workers. As a consequence, IWC organizers must invest significant time seeking out alternative sources of funding. Since most of those funds are project-based, there is a need to create projects that fit within the mandates of foundations or other funding bodies, which have pre-defined priorities.

Another challenge is that most sources of funding require a significant level of reporting and concrete deliverables, which can be difficult for grassroots organizations that emphasize long-term social transformation. Leadership development, relationships and base-building are difficult to quantify. Moreover, these funding-related challenges shift the focus of full-time organizers away from their core mandate. Year-to-year funding timelines further inhibit strategic planning, rendering IWC organizers themselves precarious.

Even in the face of these constraints, IWC remains committed to its focus on temporary, foreign and non-status workers i.e., those outside the purview of state-funded service providers and those who cannot file complaints to state agencies. IWC is one of the few organizations supporting this highly precarious population, but due to limited staffing and resources, there are tensions between prioritizing casework (an individual form of advocacy) and broader political mobilization. IWC organizers face challenges in combating precarious work conditions because, to quote Power et al. (2021: 94), “they are so exhausted labouring under them.”

One potential source of funding that could address some of these challenges is SACAIS. This funding allows for independent rights advocacy by community organizations and is accessed by neighbourhood-based tenants’ rights organizations. Significantly, the program provides core - rather than project-specific - funding. The IWC adapted their auditing practices and reporting measures in order to become eligible for the program, and after some time, the organization became part of a pool of eligible applicants. However, they still have not been successful in securing the funding, as there are presently a fixed number of recipients and no new organizations have been allocated these funds (Interview, IWC organizer). According to an IWC board member, this funding regime leaves behind “organizations that [address] structural changes in the economy … [and] changes in immigration and poor workers who are no longer part of trade unions” (Interview). More generally, such challenges underscore how neoliberal logics influence the broader funding landscape for non-profits.

## Implications of distinct institutional configurations for agency

The cases above cast light on the non-profit ‘shadows’ that are increasingly at the forefront of serving marginalized populations. Both organizations offer programs that promote enhanced inclusion by addressing the inter-locking challenges (within and beyond the workplace) that participants face. In the case of PM, this includes accompaniment, social supports, participation in policy debates, as well as the provision of hard skills. In the case of IWC, this is centred on advocacy that includes individual casework and wider campaigns, alongside accompaniment and worker empowerment.

In realizing their mandates, both organizations are clearly operating within the context of a neoliberal state apparatus, a trend most evident in the prevailing funding regime that is centered on service provision and short-term, concrete deliverables. Yet, in alignment with a relational perspective (Baker and McGuirk, 2021; DeVerteuil et al., 2020; Power et al., 2021; Trudeau, 2008), the cases present sites that are neither wholly or uniformly conditioned by the state nor the broader apparatus. Rather, the organizations represent hybrid amalgams that can exert agency through strategic and dynamic actions and collaborations (at various scales) in their efforts to realize their respective mandates. In particular, strong ties to participants and community organizations, as well as mixed sources of funding, give them insight and credibility, and hence, legitimation and leverage vis-a-vis a state that has abandoned some of its core welfare responsibilities **(**Baker and McGuirk, 2021).

However, owing to their distinct institutional configuration of funders and partners, the organizations pursue their mandates through different mechanisms and to different effect. In the case of PM, the state is an ‘institutional anchor’, due to the formal contracts and extensive funding received. This affords PM a level of stability from which to sustain accompaniment and social supports. However, it also presents challenges to programming (e.g., placing emphasis on applied training and successful placements over accompaniment or community building) and to participant selection (e.g., restricting eligibility to a finite number of migrants with status), thereby constraining the room for strategic manoeuvring in relation to these areas (Chouakri, 2019). Such interventions are bound by the government-sanctioned ‘rules of the game.’ PM’s distinct form of state-non-profit entanglement also delineates the range of interventions for countering labour market precarity, circumscribing their agency. Working within the Collectif offers a pooling of resources and participation in initiatives that can influence the social policy agenda. However, most of the organization’s socio-political functions occur through government-sponsored committees and roundtables. They attain a ‘seat at the table’ and direct ‘voice’ with respect to policy development. Yet indirect involvement in more adversarial forms narrows what Trudeau (2008) terms the ‘opportunity structure’ for redefining the rules of engagement (e.g., who is at the table and whose needs are centred).

In the case of IWC, the organization’s non-charity status and strong bonds to social movement organizations and unions - their main institutional anchors - affords them both less reliance on government funding and a greater latitude to effectively pursue their respective mandates. IWC regularly balances a mix of offerings (e.g., casework, accompaniment, empowerment workshops and campaigns). These offerings are open to all migrants, including those without status and those closed out of official state channels and services. Defining and adapting activities collectively with member participants ensures accountability to members and the constitution of the organizational space as a dynamic one (Gilmore, 2017). Their strategic positioning vis-a-vis different factions of the state, through a mix of adversarial actions as well as selective partnering, also contributes to their ability to exert influence on the ‘state apparatus’, from within and at the boundaries. What IWC gains in flexibility, however, comes at the expense of stability. To date, there is no dedicated revenue stream or core funding. Much time is needed to seek out funds, and while this may not compromise their relative autonomy in defining their activities and relations, organizers’ employment is itself precarious. This precarity limits their ability to move from incremental gains in re-regulating labour markets to sustained transformation of rules that govern policy formation (including those related to non-profit funding).

## Conclusion

Our examination of two innovative non-profit labour market intermediaries contributes to current debates on how non-profit organizations are conditioned by the state in a post-welfare context. We build on previous work on the ‘relational shadow state’ (Baker and McGuirk, 2021; DeVerteuil et al., 2020; Trudeau, 2008) to illustrate how non-profit organizations, operating within the landscape dominated by a neoliberal state apparatus, can exhibit non-linear and non-uniform relations to such an apparatus, with the ability to influence it. This is especially the case since these organizations have direct links with marginalized communities, with which their legitimacy vis-a-vis the community and state is tied.

However, our analysis is further informed by an institutional-relational approach (Jessop, 1990) to draw attention to how the institutional configuration that constitutes the non-profit space (i.e., an intersection of the broader policy and funding regime and the nature and structure of networks) mediates their agency vis-a-vis the state apparatus. In the case of PM, where the state serves a key institutional anchor and the organization is registered as a charity, there is a dedicated - though not ‘core’ - funding stream for service provision that contributes to greater organizational stability, but which can restrict agency in terms of program offerings, which participants are served and socio-political intervention. For IWC, non-charity status and institutional anchors situated at the margins or outside the state provide greater latitude with respect to defining and realizing objectives. But reliance on diverse and evolving, non-core funding creates organizational precarity in a non-profit landscape that is heavily shaped by neoliberal metrics, structuring the conditions – if not the parameters – of agency. Further analysis of the impacts of different kinds of institutional configurations for the agency of organizations with no state contracts is warranted (cf. Gonzalez Benson, 2022), as is an examination of the prospects of ‘core’ funding, which remains under-explored in the literature. The evolving practices of Canadian foundations are another significant line of inquiry, as some are increasingly supporting equity-seeking non-profits and loosening funding restrictions (Finchum-Mason, 2022).

Our analysis extends a relational view, contending that the nature and depth of agency is contingent on the organization’s distinct institutional constellation, with the organization representing a space through which a particular set of relations, resources, and funding flows. We suggest that this implies the need not only to document the manifestations of non-profit agency, but to establish the institutional dynamics which give rise to such manifestations. Such a ‘mapping’ of these configurations, we contend, can provide greater insight into an organization’s positioning vis-a-vis the prevailing state apparatus, the pathways of agency that a positioning enables and forecloses, and the potential to construct new paths altogether.

## Note

1. The authors would like to thank one of the reviewers for this point.
